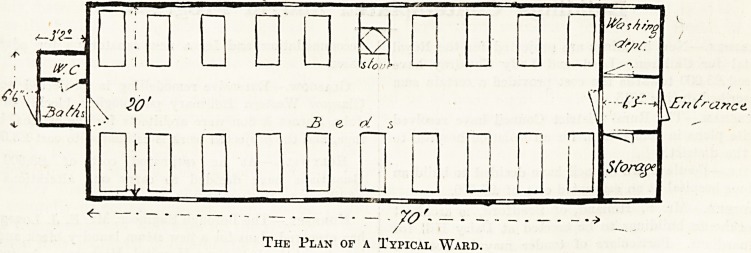# An Italian Hospital Experience

**Published:** 1912-08-10

**Authors:** Mario Ballarelli

**Affiliations:** Rag.


### HOSPITAL ARCHITECTURE AND CONSTRUCTION.

[Communications on this subject should be marked "Architecture" in the left-hand top corner of the envelope.]

### An Italian Hospital Experience.

### THE DOCKER PAVILIONS IN A MODERN HOSPITAL.

By MARIO BALLARELLI, Rag.

Up to the present the Docker pavilions have not been introduced widely in the making of hospitals. Their use has been chiefly limited either to military accommodation or to emergency wards to be utilised in case of epidemics only. However, the advantages of a pavilion system have proved to be so important and the cost of Docker pavilions is comparatively so low that one is quite justified in foreseeing for the future a larger use of this construction in many hospitals.

In 1897 the Italian Home Secretary gave to the different hospitals of the country some very severe cautions as regards the isolation of the tuberculous people from the other patients. I regret to say that before this time many Italian hospitals sheltered those suffering from tuberculosis amongst the other patients. Consequently, after the Government instructions every effort was made both by the hospital committees and by the town councils in order to comply, as far as the financial means would allow, with the ordinance.

The Hospital Committee in Genoa projected the construction of a Docker pavilion hospital as the most quick, economic, and efficacious solution of the problem. In only three months' time eight pavilions able to shelter 152 patients (95 males and 57 females) were opened. The Docker pavilion, as it has been chosen for the Genoa Tuberculosis Hospital, forms by itself a single ward 70 feet long, 20 feet wide, with an average height of 10 feet. It contains nineteen beds, thus allowing about 730 cubic feet for every bed.

The Front Elevation.

This figure may seem rather low, as Morin claims at least 880 cubic feet for every patient, and Tenon goes so far as to demand an average of 1,800 cubic feet. As a matter of fact, the calculations of J. F. Sutherland and of Lion de Fort show that in England the average space is 1,800 cubic feet per bed, whilst it is 1,600 cubic feet in France. However, we must not deduce from this that the type of Italian hospitals is less hygienic than in other countries with regard to the cubic allowance made to every bed; as, *e.g.*, the *Ospedale Maggiore* in Milan allows 3,400 cubic feet per bed.

The Plan of a Typical Ward.

The ventilation of Docker pavilions (which is carried on in a very efficacious way, as we shall see soon) may excuse the small cubic capacity. Further, when this pavilion is used in a very sheltered and sunny position (as is the case in Genoa) the possibility of keeping the windows wide open for the most part of the year withdraws all the inconveniences of a rather small cubic capacity.

The surface for every bed is about 73 square feet; this may be considered rather satisfactory, not very far from the 90 square feet claimed by Miss Nightingale as the ideal allowance in an up-to-date hospital.

The Docker pavilion consists of a wood frame (generally pinewood steeped in creosote) resting on small brick blocks about one foot from the ground. The walls consist of two thick cardboard sheets treated with asbestos and thickly varnished on both sides, leaving between them an air chamber about two inches wide, surrounding the whole of the construction. This protects the interior from the sudden changes of temperature and prevents the loss of heat.

The floor is made of narrow pinewood boards completely covered by linoleum, thus allowing of very frequent and thorough washing. The walls allow of ten windows $2\frac{1}{2}$ feet by $3\frac{1}{4}$, giving more than eight square feet of lighting surface per bed. The entrance of every pavilion leads to a small room where three other doors open, one for the ward, another to the left for the washing up of utensils, and another opposite for a small storage, entrusted to the sister-in-charge. At the other end of the ward are the w.c. and the baths. The heating in the cold season is carried on by ordinary stoves (one for every pavilion as shown in plan). These stoves are made of iron sheet lined inside with fireclay. Ventilation is obtained through four regulating openings in the roof. In the centre of the ward an electric fan is placed to assist the ventilation in calm weather.

After three years of use the pavilions were in a very good condition and they did not require much expenses of repairing. The life is calculated by the makers to be about forty years, but for practical purposes and for a provident administration not more than twenty to twenty-five years ought to be depended upon. The cost of a Docker pavilion is £500, erected and completely finished, foundations excluded, the latter being a very small item. In a sunny and dry place, like Genoa, it did not prove necessary to provide for sheltered passages between the pavilions.

The annual cost for keeping a bed in this hospital is about £48; this figure cannot be compared at all with the British hospitals, as in Italy labour, professional salaries, and foods are much cheaper than here. The 152 patients are attended by five physicians, whilst the nurses are chosen from nuns, which very unfortunately is the practice in Italy. An auxiliary staff is used for the harder work. Generally there are two attendants in every pavilion.

The Docker pavilions have proved most satisfactory as hospital wards under many points of view. They are economical, easy of transport and of erection (three days being enough for the erection of one of them). They are open to ventilation and properly protected from the extremes of temperature. They look homely, gay, and cheerful, overcoming the sad influence that the ugly, great grey buildings of many infirmaries have on the patients and their visitors. They also allow of the most careful and complete isolation. Being of one floor only and not very high they do not overshadow each other as unfortunately happens in many otherwise beautiful hospitals (*e.g.*, the Royal Infirmary of Edinburgh, where the four parallel surgical wings are so high and so near as to lead to one overshadowing the other). Owing to their simplicity they may be easily shifted or removed at any time should any alterations be required in the general plan of the hospital.

## Figures and Tables

**Figure f1:**
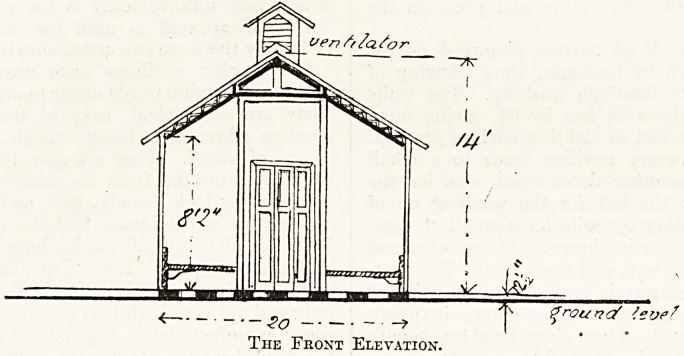


**Figure f2:**